# High-Performance Bioinstrumentation for Real-Time Neuroelectrochemical Traumatic Brain Injury Monitoring

**DOI:** 10.3389/fnhum.2016.00212

**Published:** 2016-05-19

**Authors:** Konstantinos I. Papadimitriou, Chu Wang, Michelle L. Rogers, Sally A. N. Gowers, Chi L. Leong, Martyn G. Boutelle, Emmanuel M. Drakakis

**Affiliations:** ^1^Department of Bioengineering, Imperial College LondonLondon, UK; ^2^Bioinspired VLSI Circuits and Systems GroupLondon, UK; ^3^Biomedical Sensors GroupLondon, UK

**Keywords:** analog-to-digital conversion, bioinstrumentation, biosensors, microdialysis, microfluidics, traumatic brain injury

## Abstract

Traumatic brain injury (TBI) has been identified as an important cause of death and severe disability in all age groups and particularly in children and young adults. Central to TBIs devastation is a delayed secondary injury that occurs in 30–40% of TBI patients each year, while they are in the hospital Intensive Care Unit (ICU). Secondary injuries reduce survival rate after TBI and usually occur within 7 days post-injury. State-of-art monitoring of secondary brain injuries benefits from the acquisition of high-quality and time-aligned electrical data i.e., ElectroCorticoGraphy (ECoG) recorded by means of strip electrodes placed on the brains surface, and neurochemical data obtained via rapid sampling microdialysis and microfluidics-based biosensors measuring brain tissue levels of glucose, lactate and potassium. This article progresses the field of multi-modal monitoring of the injured human brain by presenting the design and realization of a new, compact, medical-grade amperometry, potentiometry and ECoG recording bioinstrumentation. Our combined TBI instrument enables the high-precision, real-time neuroelectrochemical monitoring of TBI patients, who have undergone craniotomy neurosurgery and are treated sedated in the ICU. Electrical and neurochemical test measurements are presented, confirming the high-performance of the reported TBI bioinstrumentation.

## 1. Introduction

In the UK an estimated 238,000 people suffer from a TBI each year (Whitfield et al., 2009). Most are stabilized in an ICU at a cost of approximately £2000 per day for extended periods before discharge, often with life-changing disability. Estimates of annual UK mortality vary considerably, but it is likely that approximately 4000–7000 people die. In the US an estimated 1.7 m people suffer from TBI annually. Of these 52,000 die and 275,000 are hospitalized. A third of all injury-incurred deaths in the US relates to TBI (Faul et al., 2010) with the TBI-related healthcare cost exceeding $3 bn (Russo and Steiner, 2007; Langlois et al., 2006). Moreover, around 1,000,000 hospital admissions each year in the European Union alone are due to TBI (International Brain Injury Association). The Lancet Neurology recently termed TBI a “*silent epidemic*,” in an attempt to highlight the importance of this intracranial injury and underline the need for accurate, state-of-the-art medical systems that can assist clinicians with early diagnosis and efficient treatment (Editorial, 2010; Lingsma et al., 2010). Main role to the disability and mortalities caused by the TBI is played by an indirect, often delayed result of the primary brain injury, known as “*secondary brain injury*.” This post-traumatic complication can occur up to a week after the initial incident, which defines a clear treatment window. However, up to now, there is no robust method able to predict, when secondary brain injury begins, thus, effective timely treatment is not given.

The ability to measure minute signals reliably from the traumatized brain will allow clinical staff to detect (and perhaps ultimately predict) the onset of secondary brain injury. Combined dynamic monitoring techniques such as ECoG and real-time sampling of brain glucose by means of microdialysis—which shows that intense spontaneous waves of depolarization (SD waves) spread out from the initial site of injury into the surrounding brain tissue driving down the brain energy supply of glucose—will collect more useful sets of data from patients, resulting into a more effective treatment approach, tailored to each patient's unique condition. In order to exploit the most out of the aforementioned physiological monitoring techniques, a high-performance interfacing circuitry is required, where the signals provided by the various sensors will be processed continuously with as little noise interference as possible, thus, leading to an as high as possible signal-to-noise ratio (SNR).

The main goal of this article is to introduce a new generation of TBI monitoring instruments, capable of acquiring weak/small chemical and electrical signals from the traumatized human brain, in a low-noise, high-accuracy manner. These instruments interface with high-caliber amperometric and potentiometric biosensors, as well as with certain types of electrodes. Depending on the type of the biosensor, the output signal for a given substrate concentration is either a constant current or voltage. In biochemistry, the most common techniques used to read-out these types of biosignals with the help of (usually off-the-shelf) electronic equipments are mainly two: (i) using a transimpedance amplifier (TIA), i.e., a current-to-voltage converter (I-V) for the current input signal (generated by an amperometric sensor) and (ii) using a high common-mode-rejection-ratio (CMRR) instrumentation amplifier (V-V) for the voltage input signal (generated by a potentiometric sensor) with a specific, usually high, differential gain.

For sensitive TBI monitoring related biosignals, the aforementioned I-V and V-V conversion circuit techniques may exhibit various inaccuracies, mainly due to the strong presence of noise, once implemented on a Printed Circuit Board (PCB) with commercially available components. Unlike the conventional biosensor read-out methods, our instruments exploit the use of appropriate current- and voltage-input analog-to-digital converters (ADCs), in order to acquire and process TBI-related biosignals in a low-noise manner and immediately convert them into noise-insensitive digital signals. Comparative experimental/measured results between the traditional and the proposed techniques implemented by the new electronic platforms are presented and discussed in depth.

The article is structured as follows: Section 2 offers a brief, but inclusive introduction to the dynamic monitoring materials and methods of secondary brain injury detection. The various types of sensors been used in each monitoring category will be also illustrated, accompanied by the appropriate information regarding their operation. Moreover, in the same section the reader can find a detailed discussion of the proposed electrical and neurochemical biosensor interfacing data acquisition electronic platforms. Section 3 deals with the presentation of the various experimental results obtained from the different types of biosensors. Finally, the paper concludes with Section 4, where a detailed discussion can be found accompanied by the analysis of the measured data and their significance in TBI patients monitoring. Moreover the limitation of the specific instrumentation are discussed and envisaged future applications are proposed.

## 2. Materials and methods

### 2.1. Detection of possible causes of secondary brain injury

Brain injuries should not be treated as single events, but as complex, subsequential processes which contain multiple interplays of different effects, such as structural damage, regional ischemia, inflammation, and metabolic crisis (Obrenovitch and Urenjak, 1997; Holmin et al., 1998; von Oettingen et al., 2002; Vespa et al., 2005; Kurland et al., 2012). Without effective treatment and a complete understanding of the traumatic brain injury, the pathological features of TBI will cause patients to suffer from secondary brain injuries, which may be even more damaging. Approximately 40% of patients admitted to hospital with TBI will suffer from secondary brain injury (Narayan et al., 2002). Commonly used covariates, such as age, pupil activity and motor score, can only explain 30% of the variance in TBI outcome (Murray et al., 2007). To identify patients most at risk of developing secondary brain injury, we suggest a monitoring system that will combine results from both the electrical activity, using ECoG, and the neurochemical recordings by using biosensors coupled to a microdialysis probe. Currently, a “*wait and see*” approach is taken with any secondary injury managed after its development. CT and MRI scans provide information about the brain tissue at that particular moment in time but fails to indicate which patients are likely to suffer from secondary brain injury. A multi-modal bedside system will provide valuable information to the clinicians about the brain tissue directly and continuously over the monitoring period. Accessing this information, allows clinicians to act prior to the development of secondary brain injury and potentially will provide a more personalized approach to clinical care.

#### 2.1.1. Through electrical activity recordings—ECoG method

Spreading depolarizations (SD) waves were first identified in 1944 by Leao [Leao (1944)] and the presence of SD waves in brain injury patients has since been identified as a good predictor of secondary brain injury and poor patient outcome (Hartings et al., 2011). SD waves are the outcome of mass neuronal and astrocytic depolarization that mainly originate from ischemic brain injury and propagate across the cerebral cortex. These types of waves occur frequently to patients who are undergoing a craniotomy, which is often required when a sub-arachnoid hemorrhage (SAH), TBI, malignant hemispheric stroke, or spontaneous intra-cerebral hematoma is taking place (Strong et al., 2005; Dohmen et al., 2008; Lauritzen et al., 2011; Woitzik et al., 2011). A reliable method to detect and characterize SD waves can be provided by ECoG and is seen as a slow and huge negative potential drop, which silences the electrical activity of the cortex. Compared to typical electroencephalography (EEG) signal recordings, which are acquired from the scalp (and therefore are attenuated due to the low conductivity of bone), the ECoG signal is captured directly from the exposed surface of the cortex and thus, offers much higher spatial resolution. The approximate spatial resolution of ECoG monitoring is 1 cm with a temporal resolution of 5 ms (Asano et al., 2005).

**Figure 1:**
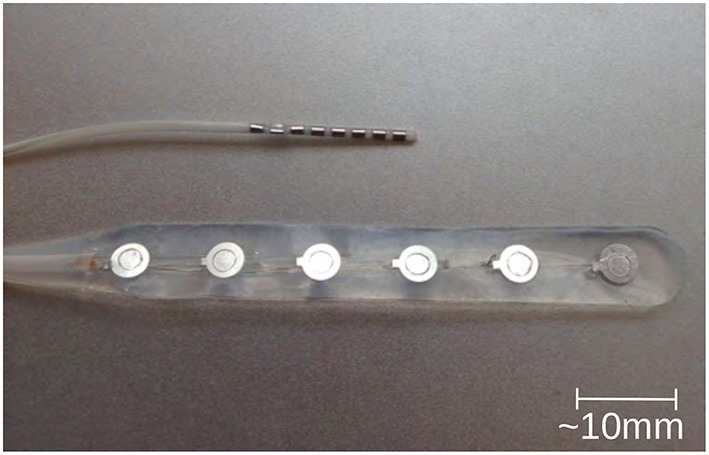
**Typical depth (upper one) and strip (lower one) electrodes, commonly used in ECoG method for TBI (photo kindly provided by Dr. Toby Jeffcote)**.

In terms of detecting and monitoring the ECoG signal, two different types of electrodes have been reported in the literature, presented in Figure 1; the strip electrode (Strong et al., 2002) and the depth electrode (Jeffcote et al., 2013). The strip electrodes are placed directly onto the cortex of the brain when the patient undergoes the craniotomy, and therefore this technique, and hence the monitoring of SD waves, is restricted to those patients who require craniotomy. Whereas, the depth electrode array allows the ECoG signal, and SD waves, to be monitored via a burr hole to the cortex. This technique does not require patients to undergo major surgery and the burr hole procedure can be conducted on the intensive care ward. This method facilitates the monitoring process and the number of available patient samples.

The cortex SD (CSD) wave that is recorded by the ECoG signal is characterized by a *low* and a *high* frequency range. In the low-frequency range of the ECoG, almost direct current (DC) range, the CSD can be seen as a negative potential shift, while in the high-frequency range of the ECoG, CSD causes silencing of spontaneous activity (spreading depression). The vast majority of clinical studies have used an alternating current (AC)-ECoG setup in the ~0.5–100 Hz range (“*high*” frequency range). In practice, AC coupling requires the transition of the raw ECoG signal through an ordinary capacitor, of appropriate value. The AC-coupling of the ECoG signal generates a high-pass filter (with behavior corresponding to the time-constant dictated by the capacitor) which eliminates the DC component of the raw ECoG signal. On the other hand, a DC-ECoG signal allows both frequency band components of the CSD wave to pass and subsequently be measured by appropriate data-acquisition systems. DC-ECoG measurements are more favorable compared to AC-ECoG data, since more information about the traumatized brain is included in the signal. Indicative publications of AC-ECoG and DC-ECoG measurements can be found in Hartings et al. (2009) and Oliveira-Ferreira et al. (2010), respectively.

#### 2.1.2. Through neurochemical changes recordings

Previous work has led to the development of a rapid-sampling microdialysis technique (rsMD) to measure brain tissue glucose and lactate levels in real-time (Parkin et al., 2005). This work involves the use of a microdialysis probe to sample the tissue in which it is placed. Traditionally, the liquid sample is collected in a vial, which is inserted into a bedside analyser by hand every hour. The analysis component of rsMD, however, connects to the microdialysis probe via a long, fine bore connection tubing and analyses nanoliter samples, online, every minute. The results from the use of rsMD in both surgery (Bhatia et al., 2006) and whilst monitoring on the ICU (Feuerstein et al., 2010) have proved that there are key neurochemical changes, which are detrimental to the brain tissue outcome that occur on this time scale.

rsMD consists of a mechanical valve connected to electrochemical detectors for glucose and lactate. There is a need for a chemical marker of depolarization as the electrical probes (electrodes) and the microdialysis probes cannot measure the same space within the tissue. Therefore, there is an error in time aligning the data and currently it is based on the approximate location of the probes as seen on scans such as MRI. The analysis equipment, plus all of the associated equipment, are placed upon a large trolley that can be wheeled to the patient when required and ideally, the trolley and the associated equipment need to be smaller to be practical in a busy ward and theater. Also, the connection tubing means that there is a time delay from sampling to analysis (rsMD takes approximately 15 min, due to low flow rates) and dispersion occurs, smearing out any fast sharp changes and increasing the difficulty in analysis. Therefore, work has been undertaken to miniaturize the system and address these key problems.

**Figure 2:**
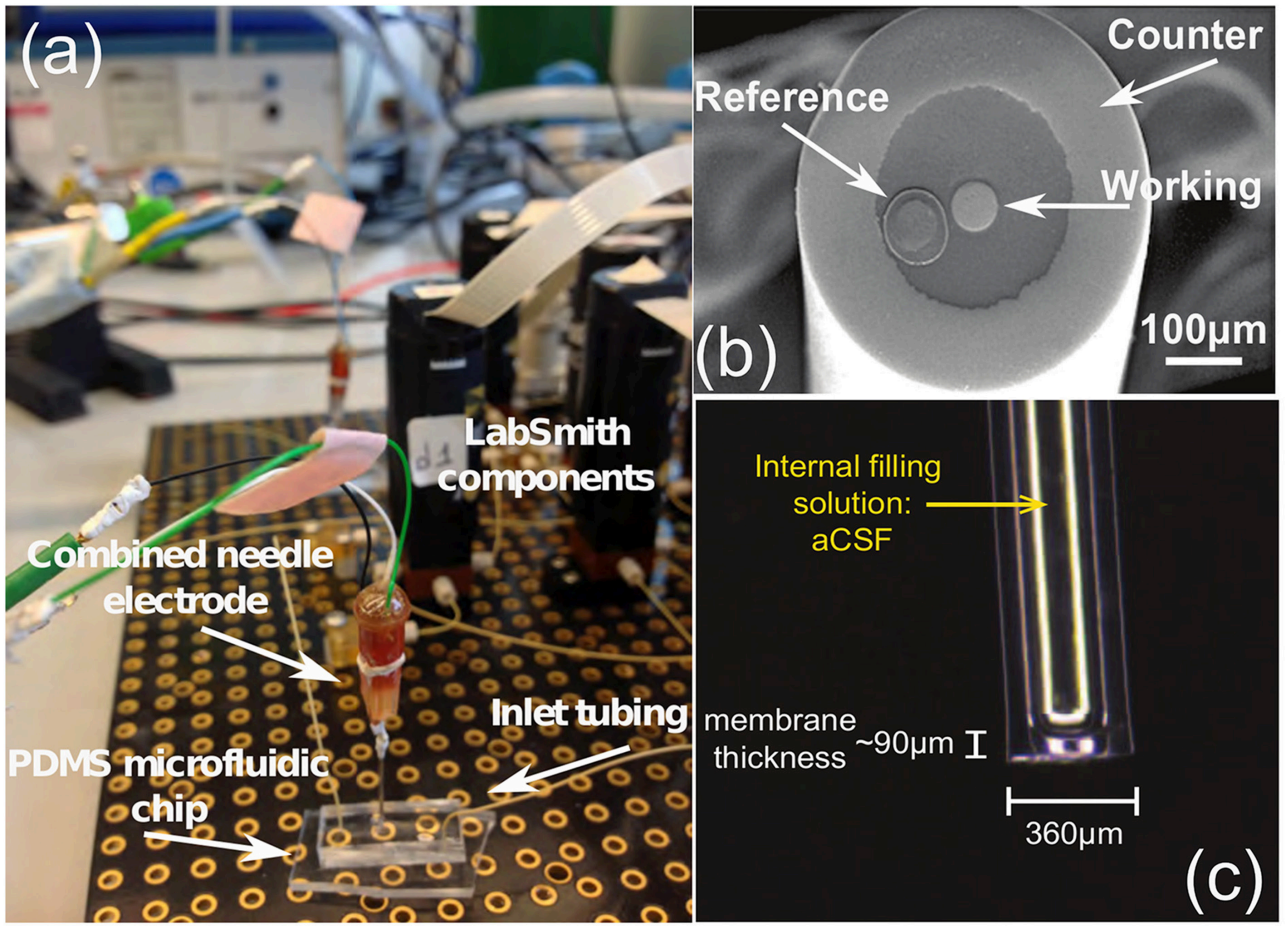
**(A)** The glucose biosensor setup, **(B)** the tip of the needle electrode and **(C)** a potassium ion selective electrode (ISE), all produced by the Boutelle Lab. The LabSmith components (also known as “uDevices”) are including valves, syringe pumps, manifold (used to control the valves), and the platform (provides interconnections of these components and the host PC). The PDMS chip is applied to convey the dialysis samples (glucose, lactate, etc.) in the microdialysis tube to the sensors, using microfluidic techniques. Finally, the combined needle integrates the three-electrode system in a single tip. The difference between an electrode and a sensor lies within the application of different enzymes. **(A)** Combined needle electrode placed onto a PDMS microfluidic chip within the LabSmith automated system. **(B)** Combined needle with three electrodes. **(C)** Potassium ISE.

The application of microfluidic platforms to microdialysis use is a recommended one. Microfluidic platforms can easily handle the nanoliter-sized samples gained from microdialysis and will miniaturize the analysis system. The use of digital microfluidics, i.e., the addition of an immiscible oil flow to segment the dialysate into tiny droplets, eliminates dispersion as droplets do not mix together, and the faster flowing oil reduces the time lag to seconds without affecting the flow rate through the microdialysis probe (Rogers et al., 2011). Smaller electrochemical biosensors for glucose and lactate, and ion selective electrodes (ISE's) for potassium, a chemical marker for depolarization, have been tested for such a purpose (Rogers et al., 2011, 2013b). Indicative types of the aforementioned biosensors and experimental setups are shown in Figure 2.

SD waves have a clear neurochemical effect on the tissue. There is a wave of hyperaemia associated with the depolarization events (Leo, 1944). The depolarization of all the cells in the local area, places a huge demand on energy sources to repolarize the cell membranes. It has recently been shown in a translational study (Rogers et al., 2013b) that the energy demand to repolarize, causes local glucose concentrations to decrease and that this demand is not met by local blood flow due to the hyperaemic wave passing the cells during the depolarization stage. This deficit in glucose availability forces the cells into anaerobic respiration and hence the concentration of lactate increases. The lactate to glucose ratio has been shown to be a good indicator of ischemia and tissue health (Rogers et al., 2013a).

In patients, monitoring the levels of glucose and lactate in the nearby at risk tissue of TBI patients using rsMD has shown that the glucose levels decrease and the lactate levels increase with corresponding evidence of SD waves (Feuerstein et al., 2010). It has been hypothesized that the local pools of glucose are slowly being diminished with each event, possibly falling to an insufficient level to sustain the cells. It is this effect which may be attributing to the growth of ischemic tissue and secondary injury in the surrounding tissue (Feuerstein et al., 2010).

### 2.2. Next generation of neurochemical and ECoG instrumentation for TBI monitoring

Potentiometric sensors, such as the potassium ISE, can in practice be considered as constant voltage sources in the *mV* range connected in series with a source resistance, which strongly depends upon the fabrication and configuration of the sensor. A typical value of the potentiometric source resistance ranges between 10^6^−10^9^ Ohms. On the other hand, a typical amperometric biosensor can be considered as a high-impedance picoampere (*pA*) to nanoampere (*nA*)-level current source (Yue et al., 2008). Thus, the input impedance levels of the sensor interface circuitry should be selected appropriately.

For potentiometric sensors, the values of their output signal range between few hundreds of μ*V* up to few hundreds of *mV*. Miniaturized TBI amperometric sensors usually provide an output current signal that ranges between some tenths of *pA* up to few *nA*. For amperometric sensors, a conversion of the sensor current input signal into a voltage one using a TIA stage is very common, while for potentiometric sensors, the use of instrumentation amplifiers of fixed/tunable gain that produce an output signal that it is generated by a potential difference between the input and a reference signal is also trivial. It should be stressed that for such current levels, the effect of the thermal noise generated by the usually high in value feedback resistor (typically hundreds of *MOhms* up to several *GOhms*) of the traditional transimpedance setup, compromises the integrity of the recorded amperometric biosensor signal, despite its very low bandwidth (Glaros and Drakakis, 2013).

**Figure 3:**
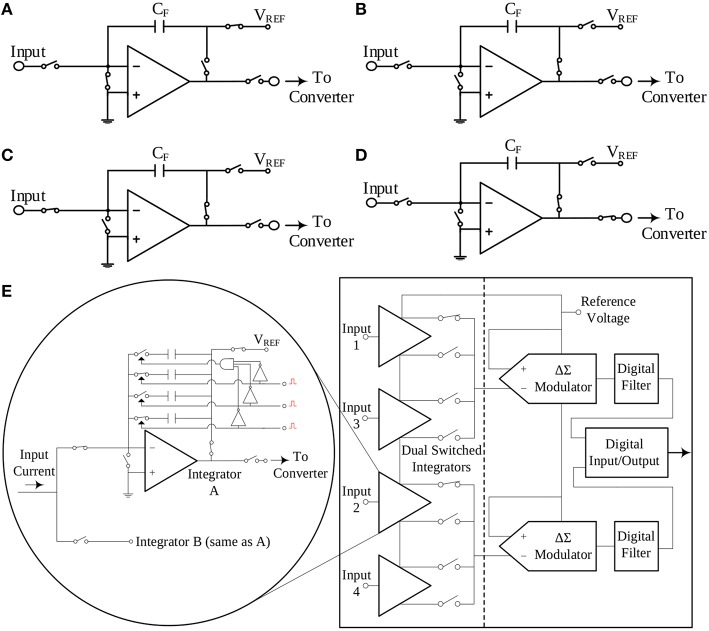
**The switched-capacitor technique exemplified by four typical configurations of a typical switched-capacitor circuit**. Proper ON/OFF combinations of the five different switches illustrated in the Figure, result to a different state of the circuit. Panel **(E)** provides a simplified block diagram configuration of the current-input ADC, which exploits the switched-capacitor architecture and has been selected to interface with the amperometric biosensors. **(A)** Reset configuration. **(B)** Wait configuration. **(C)** Integrate configuration. **(D)** Convert configuration. **(E)** A simplified architecture of the DDC family chip.

The impact of noise upon the measurements can be reduced by introducing analog or digital filtering. However, in general, filtering may also affect or even eliminate (depending on the type, order and band of operation) from the finally recorded data clinically crucial physiological information present in the noisy raw signal(s). TBI-related biosignals contain crucial physiological information in their “*raw form*.” In order to tackle the effect of the thermal noise of the resistor of a TIA setup upon the amperometric sensor current signal, the switched-capacitor paradigm has been adopted (Liu, 2006), where the input current charges a capacitor, whose charging and discharging phases are controlled by a combination of switches in the recording circuit. This technique does not call for an ohmic resistor. Hence, the integrity of the input current is not compromised by the significant levels of thermal noise due to the high-valued transimpedance resistors. In Figures 3A–D, the interested reader can find four typical configurations of a characteristic switched-capacitor recording topology.

An off-the-shelf integrated circuit chip that combines the aforementioned switched-capacitor technique with a high-precision, current input ADC is the DDC family chip series produced by Texas Instruments. For the design of our TBI boards, the quad-channel DDC114 chip has been selected. These 20-bit current-input ADCs provide adjustable full-scale ranges and integration times that allow currents from hundreds of *fA* up to few μ*A* to be measured with sufficient precision. Moreover, the dual-switched integrator front-end of this chip series, allows for continuous measurement (current integration) of the input current signal, since while one integrator is being digitized by the chip's ADC, the other one is integrating the input current. A simplified architecture of the DDC family chip is illustrated in Figure 3E.

With the use of the switched-capacitor technique for amperometric sensor measurement, we reduce drastically the presence of noise in the recorded amperometric data. For the voltage signals originating from the potentiometric ISE and ECoG electrodes presented in Section 2.1, another off-the-shelf, low-noise, high-accuracy integrated circuit solution from Texas Instruments has been chosen to interface with them, the ADS1298 family chip. Comprised of highly flexible input multiplexers per channel that can provide many configurable signal switching options, these fully-differential, multichannel, simultaneously-sampling, 24-bit resolution delta-sigma (ΔΣ) ADCs are accurate, programmable chips that can easily detect minute potential changes and record them efficiently without introducing excessive noise or offset. The differential signal of each of the 8 channels of the ADS1298 benefits from a programmable gain amplifier (PGA), while high-resolution ΔΣ ADCs follow. The high precision of the specific chip in conjunction with its high accuracy properties leads to low-noise high accuracy measurements even for very small input signals (in the range of few μ*Vs*).

With the help of the aforementioned current/voltage input ADCs, an alternative high-performance approach to the read-out of TBI-related biosignals has been adopted. Immediate digitization of the analog biosignal allows us to further process the captured signal by digital means, and therefore, without minimal corruption by the various noise sources of the environment (Park and Mackay, 2003).

#### 2.2.1. Neurochemical biosensor interfacing circuit board

**Figure 4:**
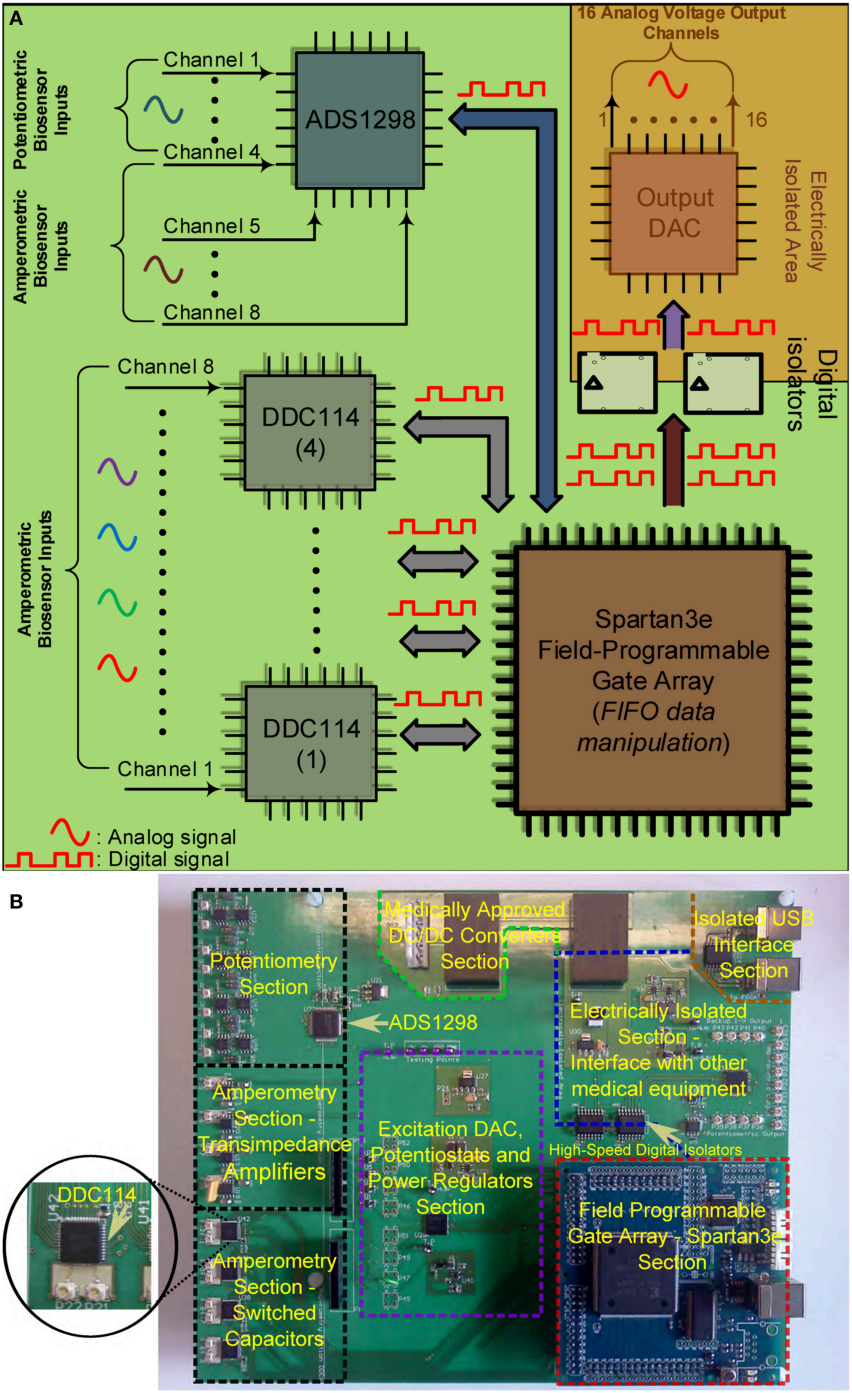
**The TBI neurochemical biosensor interfacing circuit board**. In **(A)** a simplified architecture diagram of the board can be seen, while in **(B)** the actual, fabricated electronic platform is shown, with its distinct compartments annotated on the Figure. **(A)** Operational block diagram of the TBI neurochemical data acquisition platform. **(B)** The neurochemical board with its eight distinct compartments.

Figures 4A,B illustrate the operational architecture and the physical realization of the neurochemical TBI board. Three different biosensor front-end interfacing areas have been realized: (a) the amperometry section comprising four current input, ADC chips of the DDC family, each one responsible for the monitoring of mainly two different types of biosensors, i.e., glucose and lactate biosensors. The four current-input ADCs can support the high-performance, low-noise, simultaneous interfacing with eight in total amperometric biosensors; (b) the TIA section, which offers conventional I-V read-out of up to four amperometric biosensors, generating currents in the range of tenths of nAs. The TIA interface comprises low-input bias and offset current-opamps and high-valued, ultra-high-precision resistors. The input bias current of the high precision amplifier is in the range of few femptoamperes (fAs); (c) the potentiometry section enabling the interfacing with up to four potentiometric biosensors. The potentiometry channels involve low-offset, high-input impedance opamps. Eight in total (four corresponding to the TIA section and four to the potentiometry section) interfacing channels, whose output is of voltage form are converted by the aforementioned voltage-input ADC chip (see Figures 4A,B). Referring to the same Figures (see bottom right side of Figure 4B), the Spartan3e series FPGA from Xilinx has been employed for the control and programming of the seven in total converters [ADCs and Digital-to-Analog converters (DACs)] of the board as well as for the Digital Signal Processing (DSP) of the digital data. The FPGA is clocked from a 64 MHz clock module and can be programmed via USB2.0 interface. DACs are used (see center of Figure 4B) for the controlled and programmable generation of the appropriate reference electrode voltage levels. Bearing in mind the regulations governing medical instrumentation, medically approved powering-up modules (AC/DC and DC/DC converters) are employed (see top of Figure 4B).

The last but not least compartment of the neurochemical board is the electrically isolated area located at the top right part of the board (see Figure 4B). This area is protected from the rest of the board by the existence of a physical gap of 2 mm drawn around every power plane of the board, while all the digital lines that connect the FPGA with the output DAC that is projecting the measured input signal are passing through two 5 kV RMS quad-channel, high-speed digital isolators. This section of the board can be seen alternatively as a separate PCB “*nested*” inside the neurochemical board, since there is no electrical connection between this section and the rest of the board. This section is operating with its own power and ground planes. The “*nested*” PCB aims at minimizing digital switching noise to the rest of the board, while also contributing to the patient isolation. A USB digital isolator has also been employed, in order to electrically isolate the board from the patient/user.

The board comprises eight different signal and power layers/planes with the front-area of the top layer—which hosts the DDC amperometry, the TIA amperometry and the potentiometry section (see Figure 4A)—been fabricated using Rogers *RO*4350*B* material, an advanced PCB material characterized by and ultra high resistivity of 10^12^*Ohms*, instead of the typical FR4 material. *RO*4350*B* minimizes any leakage currents between the sensor outputs and the converters. In addition appropriate guard rings have been placed around the input connectors interfacing with the biosensors. The board has been designed with a certain level of intentional redundancy. Referring for example to Figure 4A, it can be seen that the collected digitized data are output through an appropriate DAC as analog channels (shaded part of Figure 4A). This allows for the interfacing of the proposed board with visualization suites (such as Powerlab®) enabling the time-aligned, concurrent illustration of dynamic physiological data. Table 1 summarizes the characteristics of our neurochemical board for TBI.

**Table 1:** **Electrical characteristics of the neurochemical TBI board**.

	**Sections**		
**Characteristics**	**Sw. Cap. Amperometry**	**T.I.A. Amperometry**	**Potentiometry**
Number of input channels	8	4	4
Resolution (bits)	16/20	–	24
Data rate (kSPS)	3.125	–	0.5–32
Input bias current (pA)	0.1	0.003	0.2^*^
Total analog current supply (mA)	~ 56	~ 5.2	~ 15.1
Total digital current supply (mA)	~ 2	–	~ 0.5
Number of analog output channels		24	
Board dimensions (cm)		23 × 19	
Medically graded		Yes - using power/digital signal/USB isolators	
IC controller		Spartan3e - 64 MHz - FPGA	
Power supply		220V AC, 50 Hz	

* *This is the input bias current of the analog circuitry before the voltage-input ADC*.

#### 2.2.2. Electrocorticography (ECoG) circuit board

**Figure 5:**
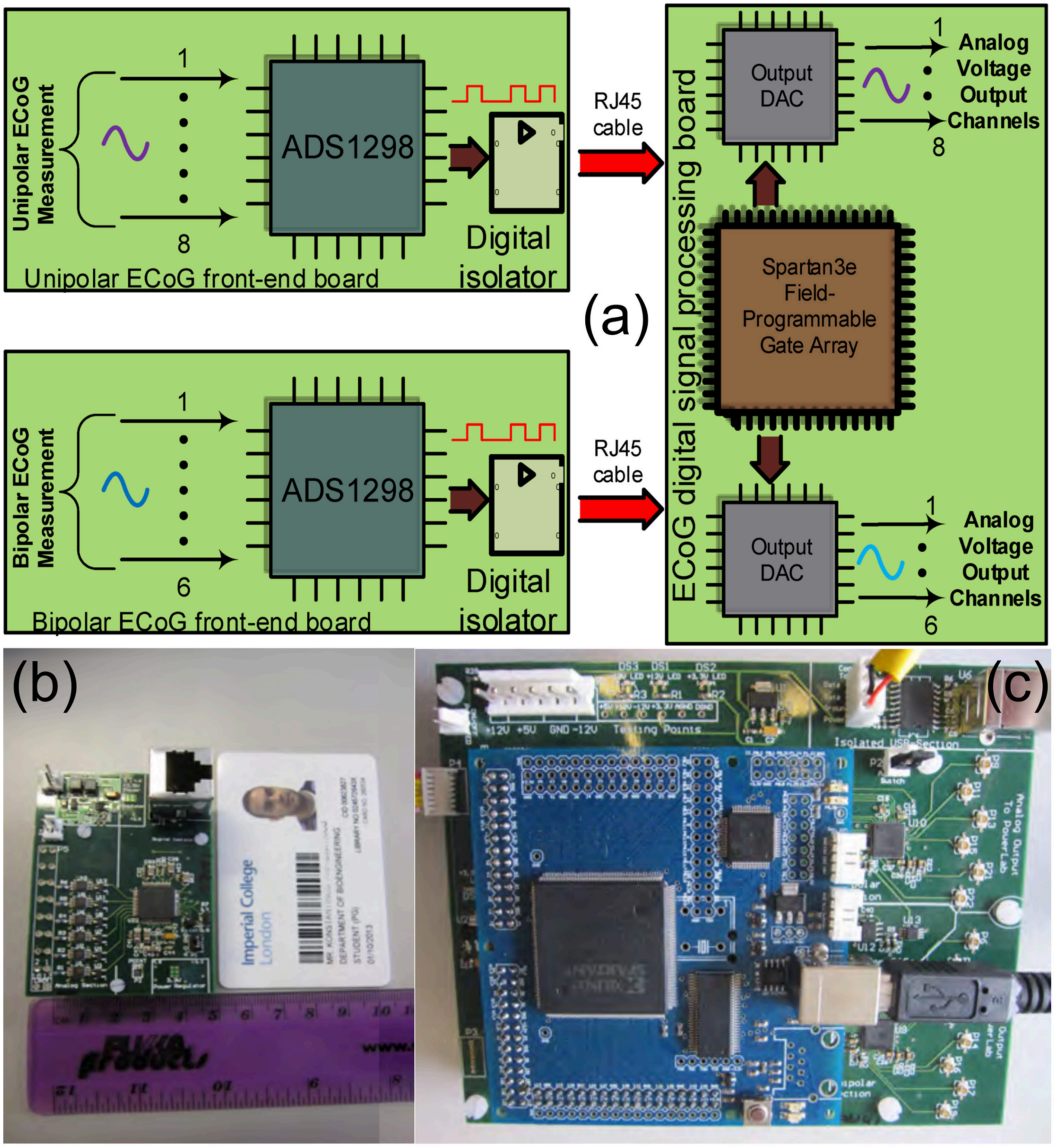
**The TBI ECoG electronic platforms for the accurate detection of minute electrical brain activities**. **(A)** Operational block diagram of the TBI ECoG data acquisition platforms. **(B)** 6-channel ECoG front-end board. **(C)** The ECoG digital signal processing board.

The ECOG platform comprises three different boards: the digital signal processing board and two front-end boards, a unipolar and a bipolar one (see Figure 5). These two front-end boards have practically the same architecture, however, they perform different types of ECoG measurements. When the depth electrode is used for an ECoG measurement, unipolar measurements take place, where the eight channel inputs from the depth electrode are subtracted from a ninth reference electrode, located at a specific area of the scalp. When the strip electrode is used, bipolar measurements take place, where the six signals/channels of the strip electrode are subtracted in a sequential form.

Figure 5A shows the operational architecture of the ECoG data acquisition platforms. Figures 5B,C illustrate the fabricated ECOG boards. The front-end board shown in Figure 5B is a bipolar ECoG board: six low-power, high-precision, non-inverting amplifiers and voltage buffers “feed” the ADS family chip, which converts the collected ECoG data into digital signals (the reader should note that the unipolar board shown in Figure 5A has similar architecture). Both the bipolar and the unipolar boards are connected to the digital signal processing board by means of RJ45 cables, through high performance digital signal isolators, which ensure the patients protection from any unwanted currents or voltages. The digital signal processing board comprises a Spartan3e FPGA and two eight-channel output DACs, which enable the real-time visualization and post-processing of the collected ECoG data by mature clinical monitoring modalities, such as Powelab®.

The “*splitting*” of the ECoG monitoring system into two parts favors noise reduction and preservation of the signal's integrity. The front-end board has been designed to be battery-operated (which further favors noise reduction compared to power-supply powered solutions) and to be placed very close to the patient's head (ideally on the scalp), while the digital signal processing board can be placed relatively far away from the patient (~ 2 m away), thus, the amplification and immediate digitization of the data takes place close to the patient, as less affected by any potential noise sources in the ICU. Subsequently, the noise-immune digitized ECoG signals are exported through the RJ45 cables to the digital signal processing board for visualization and post-processing. If the ECoG boards were not split from the DSP board, then the weak raw ECoG signals would first need to be conveyed through a 1–2 m cable (which makes them more susceptible to noise since they are still in analog form) to the combined “*front-end/DSP*” board fabricated on the same substrate and powered-up by a noisier power source. Such a solution would be detrimental to the signals of interest integrity. Table 2 summarizes the characteristics of our ECoG boards for TBI.

**Table 2:** **Electrical characteristics of the ECoG TBI board**.

	**Type of ECoG board**	
**Characteristics**	**Unipolar/bipolar front-end**	**Digital signal processing**
Number of input/output channels	6/8 (only input)	14 (only output)
Resolution (bits)	24	16
Data rate (kSPS)	0.5–32	–
Input bias current (pA)	30^*^	–
Total analog current supply (mA)	8.35/9.85	17
Total digital current supply (mA)	0.5	17
Boards dimensions (cm)	7 × 5	11 × 14
Medically graded	Yes - using power/digital signal/USB isolators	
IC controller	Spartan3e - 64 MHz - FPGA	
Power supply	5V battery	220V AC, 50 Hz

* *This is the input bias current of the analog circuitry before the voltage-input ADC*.

## 3. Measured results

The high-performance of the proposed neuroelectrochemical data acquisition platforms has been tested by means of amperometric and potentiometric experiments. With respect to the amperometric measurements, an ultra-high precision current source device (Keithley6221), an electrode for a simple reduction-oxidation experiment and a glucose biosensor have been employed (please refer to Figure 2), in order to both exemplify and quantify the performance of the proposed TBI instrument. The amperometric experiments have been performed using the neurochemical board shown in Figure 4B. With respect to the potentiometric measurements, an ISE has been fabricated, in order to detect concentration changes in a given solution (see Figure 2). The ECoG platforms shown in Figures 5B,C have been tested by employing surrogate ECoG signals, controlled by a high-precision signal generator, Agilent33220A that was able to “*play-back*” in time the artificial signal and provide it to the ECoG platforms.

### 3.1. Amperometric measurements

#### 3.1.1. Ultra-high precision current source measurements

The scope of the first experiment is to provide compelling comparative measurements of the switched-capacitor-based amperometry front-end of the proposed neurochemical board, which exploits the DDC family chip vs. a separate traditional TIA board, which was formerly used by the Boutelle Group for interfacing with amperometric biosensors. The latter board incorporates OPA129 amplifiers by Texas Instruments and 1% tolerance, 10*GOhms* nominal value resistors, which enable the read-out of current levels as low as a few tenths of picoAmperes. Both setups have been tested using the same Keithley6221-generated currents, ranging from 10 to 100 pAs.

The integration time necessary for the current signal conversion/digitization has been programmed appropriately for each current level and in accordance with the fundamental switched-capacitor equation and the specifications of the chip:

$$\begin{array}{l} i_{\text{average}}\left(t\right)=\frac{1}{T_{\text{Int}}}\int_{0}^{T_{\text{Int}∕2}}\partial q\left(t\right)=\frac{q\left(T_{\text{Int}∕2}\right)-q\left(0\right)}{T_{\text{Int}}}. \end{array}$$

The quantity *T*_Int_ denotes the total integration period for each dual switched integrator of the chip (bear in mind Figure 3 and more specifically Figure 3E).

**Figure 6:**
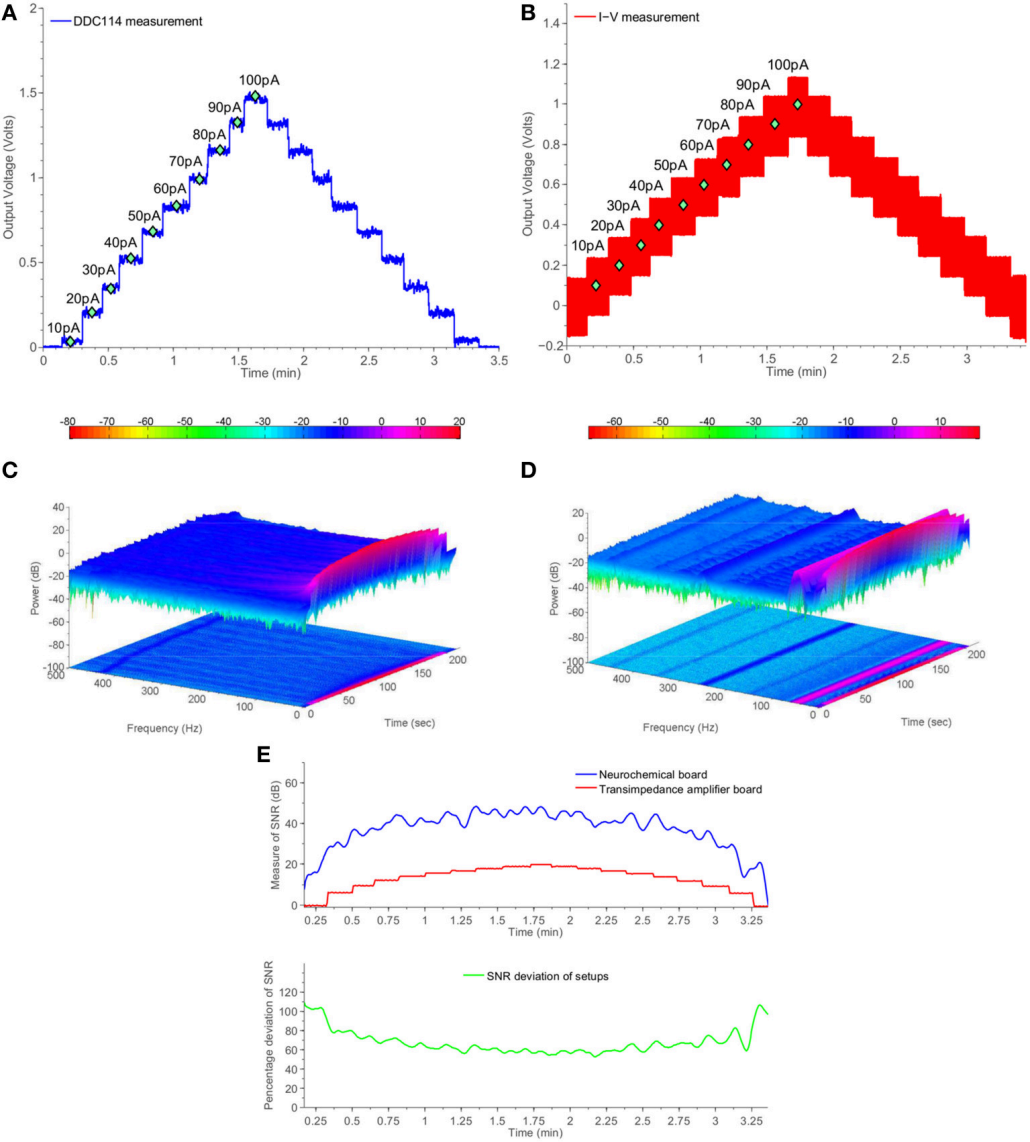
**Comparative measurement results using the switched-capacitor-based section of the neurochemical biosensor interfacing circuit board shown in Figure 4B and a common transimpedance amplifier setup**. The current input for both setups is provided by a Keithley6221 current source. **(A)** DDC measurements from Keithley6221. **(B)** I-V measuments from Keithley6221. **(C)** Two and three dimensional spectrogram of **(A)**. **(D)** Two and three dimensional spectrogram of **(B)**. **(E)** Measure of time-dependent SNR for both setups.

Figure 6 presents comparative test measurements from the amperometry section of the neurochemical board and the OPA129 TIA board. Both front-ends are interfaced with a Powelab® visualization suite. The digital code produced by the DDC114 chip and corresponding to the values of the test current input signal has been processed and projected through an output DAC module, located at the “*PCB inside a PCB*” electrically isolated part of the neurochemical board (top right part of Figure 4B). The range of the output voltage of the DAC is programmable and has been set such that it produces and output voltage level as close as possible to the one ideally expected by the OPA129 TIA setup for the given current values.

Comparing Figures 6A,B in conjuction with Figure 6E, it becomes clear that the DDC114-based amperometry front-end is characterized by higher SNR than the OPA129-based TIA setup for the whole range from 10 to 100 pAs. The SNR is improved by more than 20 dBs. Such an improvement is attributed to associate with both the absence of the large feedback resistor (and the thermal noise) and to the fact that the switching action introduced a *sinc* filtering action, scalable in the frequency domain by means of the integration time *T*_Int_. In the case of Figure 6, *T*_Int_ is programmed to be ~300 ms, which corresponds a first finite frequency notch of ~3 Hz followed by further notches.

Figures 6C,D provide two and three dimensional spectrograms for the experiments of Figures 6A,B, respectively, which confirm the absence of spurious tones in the case of the proposed amperometry front-end. Referring to Figure 6D, it becomes clear that the TIA setup suffers from the presence of several unwanted frequencies corresponding mostly harmonics of the 50 Hz tone. The combined time- and frequency-domain results of Figure 6C show that the amperometry modules of the proposed TBI instrument serve the purpose of enabling the acquisition of high-quality neurochemical data, which in turn will attribute to the safer clinical management of the TBI patient.

#### 3.1.2. Electrode measurements

**Figure 7:**
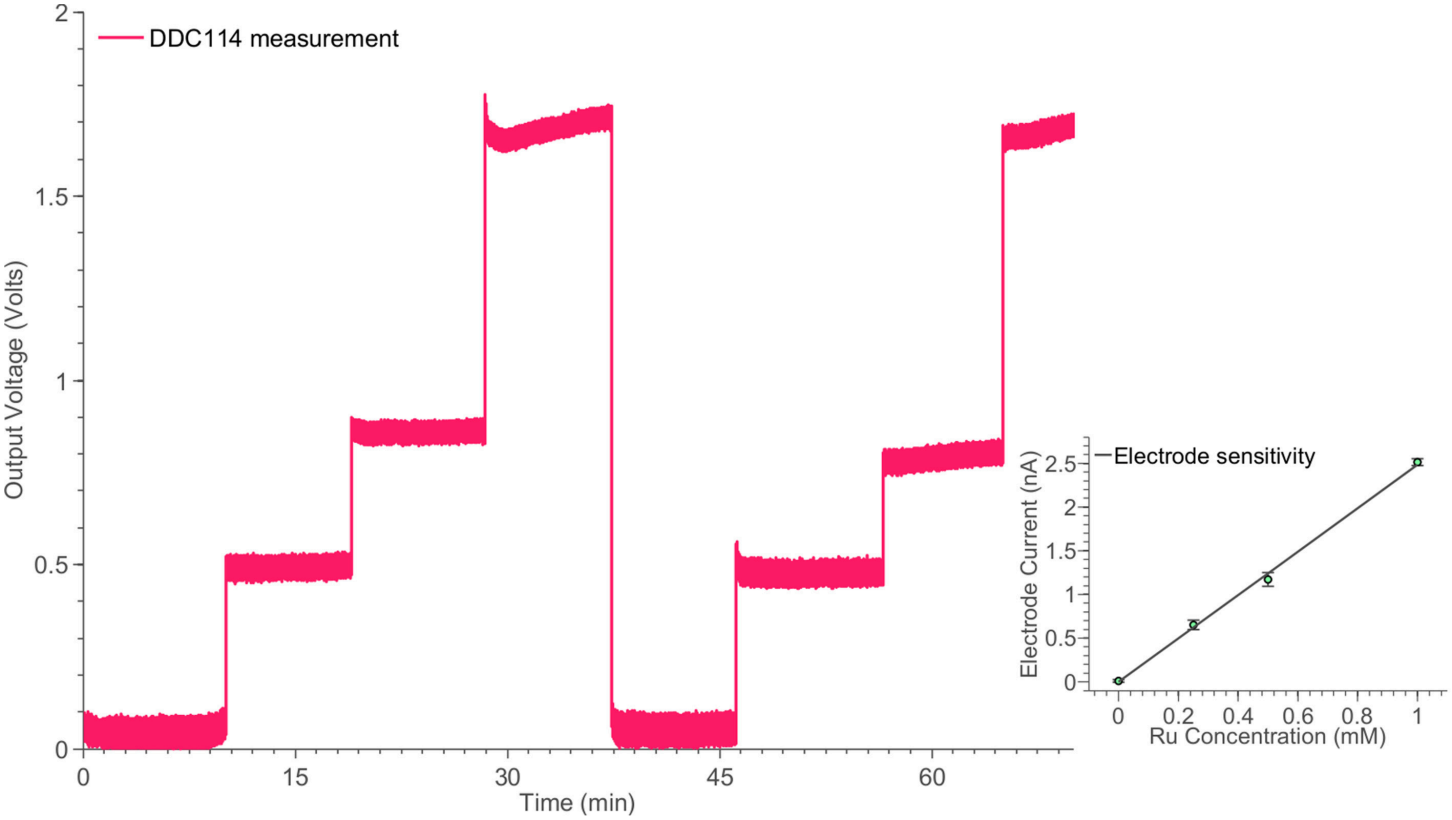
**Monitoring the time response of the electrode cell shown in Figure 2B for different ruthenium hexaamine concentrations, using the switched-capacitor amperometry section of the neurochemical biosensor interfacing circuit board, shown in Figure 4**. The linear relation between the ruthenium hexaamine concentrations and the output current of the electrode is shown in the inset of the Figure.

In order to investigate the high-performance consistency of the neurochemical board, a second amperometric experiment has been conducted. This time, a simple Redox couple has been employed, aiming at testing the neurochemical board operation in the current range from ~500 pA to ~3 nA. The electrode cell was prepared as previously described (Rogers et al., 2013b). Briefly, the method of electrode fabrication is to thread platinum and silver wires through hypodermic needles and epoxy glue was used to seal them in place. The tip of the needle was cut and then polished using alumina slurries (1, 0.3, and 0.05 μm sequentially). The platinum wire is used as the working electrode, the silver wire is chloridized to form a Ag∣AgCl reference electrode and the stainless steel shaft is used as the auxiliary electrode. Ruthenium hexaamine (Sigma) solutions were prepared at varying concentrations of 1, 0.5, 0.25, and 0 mM. Prior to taking measurements, each ruthenium hexaamine solution was bubbled with nitrogen for 20 min. The working electrode was held at a constant potential of −0.5*V* vs. Ag∣AgCl for the duration of the experiment. The constant potential has been provided by an on-board DAC and the on-board high-accuracy potentiostats (see Figure 4B). The solution was changed periodically to different concentration levels enabling the formation of the calibration curve, shown in Figure 7.

#### 3.1.3. Glucose measurements

**Figure 8:**
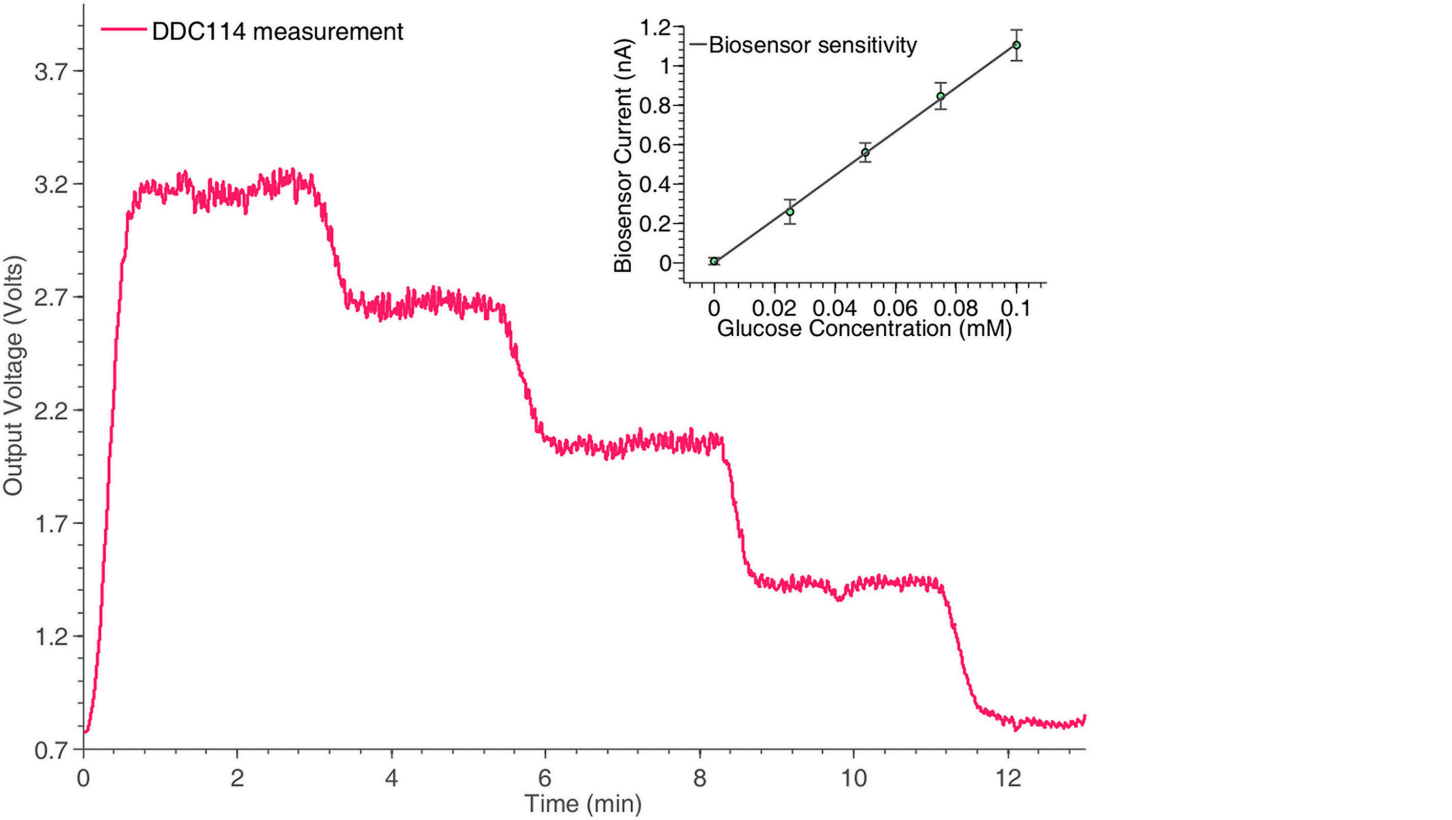
**Monitoring the time response of the glucose biosensor shown in Figure 2A for different concentrations, using the switched-capacitor amperometry section of the neurochemical biosensor interfacing circuit board, shown in Figure 4**. The linear relation between the glucose concentrations and the output current of the biosensor is shown in the inset of the Figure.

The final amperometric experiment describes the interface of a glucose biosensor used for the monitoring of TBI patients with the proposed neurochemical board. The working electrode of the sensor has been connected to the input of the DDC module, while its counter and reference electrodes have been appropriately connected to appropriately programmed on-board potentiostats. For the specific measurement, the concentration of glucose within a fake microdialysate stream was varied using an automated LabSmith microfluidic platform. Glucose oxidase (GOx)(Genzyme), followed by horseradish peroxidase (HRP) (Genzyme) dissolved into a solution containing 1.5 mM ferrocene monocarboxylic acid (Fc) (Sigma), was added into the flow stream containing different concentrations of glucose (100, 75, 50, 25, and 0 μM) and the mixture was then passed over a combined needle electrode within a microfluidic PDMS flow cell (please refer to Figure 2). To detect the level of glucose, hydrogen peroxide generated by the reaction between glucose and GOx, reacts with the HRP and Fc mixture to form ferrocinium ions, which can be detected by reduction at the electrode surface, when that is held at 0.0*V* vs. Ag∣AgCl. The results of the specific experiment are shown in Figure 8.

At this point, it should be stressed that the trend seems slightly noisier compared to the measurement of the ideal current input signal in Figure 6A, since the pumps been used to manipulate the fluid samples contributed significantly to the overall signal's noise levels. The pumping motion causes small fluctuations in the movement of the solution across the surface of the sensor, which is flow sensitive and hence disturbances in the current signal will be seen.

### 3.2. Potentiometric measurements

Apart from the previous amperometric experiments, an indicative potentiometric experiment has been also conducted with the help of the proposed neurochemical circuit. The specific potentiometric experiment implements a potassium ISE-neurochemical board interface and demonstrates the faithful monitoring of the various potassium concentration changes from the electronic platform. Moreover, in this section, a simulation of an ECoG measurement will be presented. The “*artificial*” ECoG measurement has been conducted with the help of the two ECoG boards (digital and analog front-end) presented in Figures 5B,C, respectively.

#### 3.2.1. Potassium ISE

**Figure 9:**
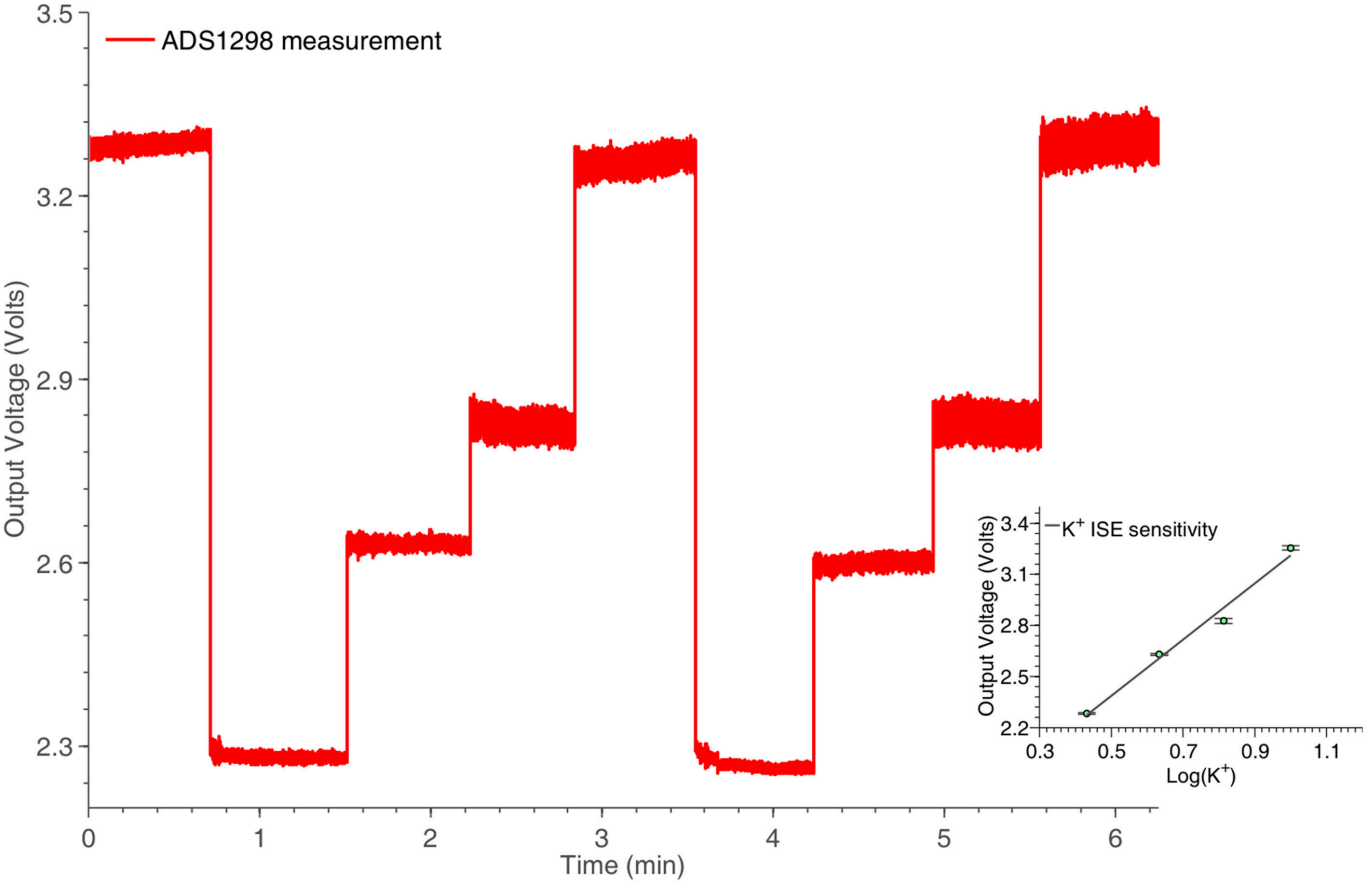
**Potassium ISE time response for various concentrations, captured by the potentiometry section of the neurochemical biosensor interfacing circuit board, shown in Figure 4**. The linear relation between the logarithm of the potassium concentrations and the output voltage of the system is shown in the inset of the Figure.

An ISE features an ion-sensing membrane that selectively binds with the ion of interest, and its output potential is proportional to the activities (or concentration) of the ion. A miniaturized potassium ISE had been developed and optimized in the group and was used in this work. It consisted of a polymer membrane casted at one end of a polymer electrode body (perfluoroalkoxyalkane tubing, o.d. 360 μm, i.d. 150 μm), an internal Ag∣AgCl reference electrode and an internal filling solution of physiological saline. The membrane consisted of 0.2 mg potassium tetrakis(4-chloropheyl)borate, 150.0 mg Bis(2-ethylhexyl) sebacate, 66.0 mg poly(vinyl chloride) and 2.0 mg of potassium ionophore. All the chemicals are obtained from Sigma-Aldrich (UK). The assembled ISEs were stored in aCSF at 4°C when not in use. Experimentally, the output voltage of the ISE was measured against an external Ag∣AgCl reference electrode (saturated at 3 M KCl). The ISEs were calibrated at 25°C and had a Nernstian sensitivity of ~59 mV/dec and a good temporal response for the physiological range of interest. The potassium ISE has been connected to one of the channels of the ADS1298, located in the potentiometric section of the neurochemical board. The results of this experiment are shown in Figure 9.

#### 3.2.2. Artificial ECoG/SD signal

**Figure 10:**
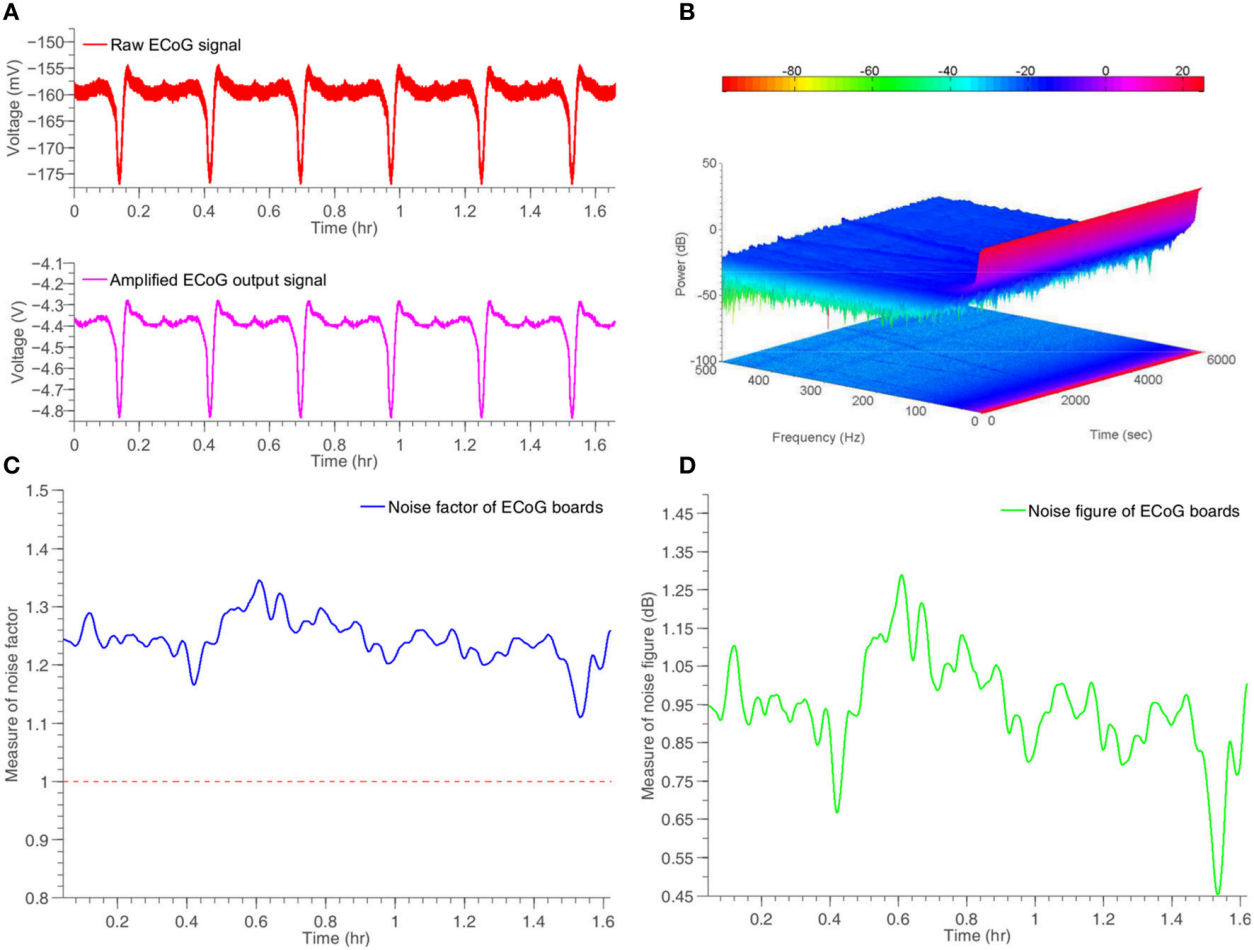
**Time response and noise performance results of a simulated ECoG/SD signal generated by Agilent33220A signal generator and recorded by the ECoG board, shown in Figure 5**. **(A)** Input and amplified output artificial ECoG signal. **(B)** Two and three dimensional spectrogram of the output signal. **(C)** Measure of time-dependent noise factor of the system. **(D)** Measure of time-dependent noise figure of the system.

The proposed ECoG monitoring system shown in Figures 5B,C has been tested by means of an “*artificial*” ECoG/SD signal. This signal has been recorded from an animal and closely resembles an actual human ECoG/SD signal. The amplitude of the specific signal is negative and ~15 mV from peak-to-peak, a value that complies with the amplitude of a typical human ECoG/SD signal. The frequency of the signal has been set to 0.1 Hz, in order to approximate the frequency of a real SD wave produced by a human injured brain. The artificial signal has been loaded to the Agilent33220A signal generator, which was fully controlled by a PC through USB interface. The whole experiment lasted for approximately 1 h and a half and included six artificial SD waves. The 6-channel front-end ECoG board shown in Figure 5B has been used to record the artificial signal and send it to the main DSP ECoG board of Figure 5C. The connection between the boards has been created with the use of an RJ45 cable. Figure 10 illustrates the indicative results obtained from a single channel of the system. The rest five channels exhibit identical behaviors.

## 4. Discussion

### 4.1. Current system limitations and future improvements

Although the neuroelectrochemical data acquisition system exhibits remarkable accuracy and very good noise performance, its high performance amperometric section with the current input ADC exhibits some limitations. This commercially available converter is able to detect very small albeit positive only input currents, i.e., currents that are “*entering*” the chip. Therefore, only reduction currents from a redox electrochemical reaction can be measured. This may limit the type of biosensors deployable with this converter. In order to tackle this issue, in a future version of the proposed neuroelectrochemical board, a custom-made, current-input ADC integrated circuit will be designed in standard CMOS process, able to detect currents flowing bidirectionally (toward and out of the system), without compromising the system's total accuracy and low-noise properties.

With respect to the ECoG analog boards shown in Figure 5B: their DC-coupled channels allow for the clinical teams to study the DC level changes while monitoring the ECoG signal. However, the very probable drift of the ECoG signals might cause the saturation of the front end electronic system. In order to prevent this from happening, a DC-level calibration circuit controller can be introduced. This controller will be able to monitor the signal's DC drift rate and adapt in real-time the system's DC levels, in order to ensure that the voltage input ECoG signal will never exhaust the maximum detection levels of the recording circuitry.

Additional modalities such as wireless transmission, linking the recorded physiological data with adverse event detection software installed on a base station can enrich the current instrumentation platform enabling the monitoring of mobilized TBI patients (usually 3 days after craniotomy surgery) and providing a more flexible approach to the early detection and subsequent prevention of unwanted patient complication in the ICU.

The inevitable reduction of biosensor sensitivity over time means that during long-term monitoring by means of the same biosensor, the same value of chemical concentration under detection will provide different electrical readings at different times. This issue, when left unaddressed, may reduce the clinical usefulness of the platform as a whole. The problem can be addressed “*head-on*” by incorporating an auto-calibration system which will determine the used sensor's varying sensitivity at specific, short intervals and thus ensure the accuracy with which the monitored concentration levels are detected during long periods of monitoring. Expectedly, the sensor calibration module can be configured and controlled wirelessly.

Finally, a new online microfluidic-based sensor system has been developed for use in combination with a microdialysis probe. The microfluidic platform is connected to the microdialysis probe via a 1 m connection tubing and sits on a trolley by the patient bedside. Each sensor is housed on a micorfluidic chip within an electrode chamber, each containing 68 nL of dialysate. The system currently continuously analyses the levels of potassium, glucose and lactate within the patient dialysate. This could, in future be expanded to monitor other key molecules of interest, such as pyruvate or glutamate.

### 4.2. Conclusions

This paper has presented the design, realization and performance of novel bioinstrumentation tailored for the collection of high-quality electrical and neurochemical physiological data from sedated TBI patients who have undergone craniotomy surgery and are in danger of suffering from secondary brain injuries.

Our multi-channel neurochemical instrument supports the collection of physiological data from both amperometric and potentiometric biosensors. It enables the acquisition of low-noise, high-performance amperometric measurements by means of rapid sampling microdialysis and microfluidics-based biosensors which can measure the injured brain tissue levels of glucose and lactate. Our test measurements have confirmed that our switched capacitor-based amperometry channels are characterized by superior noise performance when compared against classic opamp-based transimpedance stages whose performance and resolution is severely limited by the feedback resistor-induced thermal noise. Potentiometric potassium measurements further confirmed the high-performance operation of our neurochemical instrument. The collection of glucose, lactate, pyruvate and potassium physiological data has been identified recently as clinically useful for the treatment of TBI patients by authoritative consensus statements (Hutchinson et al., 2015).

Further test measurements have confirmed the ability of our new multi-channel ECoG instrument to support the collection of high-quality low-noise electrical ECoG/SD TBI data. Both the neurochemical and the ECoG instruments are compact in size and can replace the bulky (~1*m*^3^), trolley-based and impractical multi-instrument collections with multiple liquid and electrical connections currently deployed in advanced Neurotrauma ICUs and used for the monitoring of TBI patients. The new TBI bioinstrumentation presented here reduces significantly the connecting wires and tubing between the clinical team and the patient whilst they are being stabilized. Such a characteristic is important in the case of multi-trauma patients in need of emergency treatment since less clutter around the patient allows more effective the clinicians intervention. Moreover, electrical and neurochemical physiological data collected by our new TBI instrumentation will be “fed” in real-time to our adverse event detection software tailored for TBI patients which, ultimately, will alert ICU staff to intervene before the onset of secondary injury, hence allowing for the treatment of the patient in an effective and timely manner.

In the longer term we see the proposed instrumentation (and miniaturized variations of its discussed before) as being key technology for monitoring of stroke patients. This is a very large patient group who, from our animal studies and the few patients that have been monitored invasively (e.g., hemorrhagic stroke) are at risk of secondary brain injury detectable by our monitoring strategies. It is our expectation that such monitoring will define sub-populations of stroke patients who would benefit from direct neurosurgical intervention (e.g., decompressive craniectomy). In particular very little is understood of the pathophysiology of the decompressed hemisphere. A minimally invasive neuromonitoring system has the potential to revolutionize our understanding of the needs of these patients.

## Author contributions

KP: conceived and designed the experiments, designed, fabricated and programmed the electronic instruments, participated in the experiments, analyzed the experimental data and wrote the manuscript; CW: programmed the electronic instruments, participated in the experiments and wrote the manuscript; MR: conceived and designed the experiments, fabricated the biosensors, participated in the experiments, analyzed the experimental data and wrote the manuscript; SG: fabricated the biosensors, participated in the experiments, analyzed the experimental data and wrote the manuscript; CL: fabricated the biosensors, participated in the experiments, analyzed the experimental data and wrote the manuscript; MB: conceived and designed the study and wrote the manuscript; ED: conceived and designed the study and wrote the manuscript.

### Conflict of interest statement

The authors declare that the research was conducted in the absence of any commercial or financial relationships that could be construed as a potential conflict of interest.
